# Impact of COVID-19 Pandemic on Treatment Management and Clinical Outcome of Aneurysmal Subarachnoid Hemorrhage – A Single-Center Experience

**DOI:** 10.3389/fneur.2022.836422

**Published:** 2022-03-21

**Authors:** Sepide Kashefiolasl, Lina Elisabeth Qasem, Nina Brawanski, Moritz Funke, Fee Keil, Elke Hattingen, Christian Foerch, Volker Seifert, Vincent Matthias Prinz, Marcus Czabanka, Juergen Konczalla

**Affiliations:** ^1^Department of Neurosurgery, University Hospital Frankfurt, Frankfurt, Germany; ^2^Departments of Anesthesiology, Intensive Care Medicine and Pain Therapy, University Hospital Frankfurt, Frankfurt, Germany; ^3^Institute of Neuroradiology, University Hospital Frankfurt, Frankfurt, Germany; ^4^Department of Neurology, University Hospital Frankfurt, Frankfurt, Germany

**Keywords:** aneurysmal subarachnoid hemorrhage (aSAH), cerebral vasospasm (CVS), delayed cerebral ischemia (DCI), clinical outcome, COVID-19, pandemic, healthcare system

## Abstract

**Background:**

Previous studies reported decreased volumes of acute stroke admissions during the COVID-19 pandemic. We aimed to examine whether aneurysmal subarachnoid hemorrhage (aSAH) volumes demonstrated similar declines in our department. Furthermore, the impact of the pandemic on disease progression should be analyzed.

**Methods:**

We conducted a retrospective study in the neurosurgical department of the university hospital Frankfurt including patients with the diagnosis of aSAH during the first year of the COVID pandemic. One year cumulative volume for aSAH hospitalization procedures was compared to the year before (03/2020 – 02/2021 vs. 03/2019 – 02/2020) and the last 5 pre-COVID-pandemic years (2015-2020). All relevant patient characteristics concerning family history, disease history, clinical condition at admission, active/past COVID-infection, treatment management, complications, and outcome were analyzed.

**Results:**

Compared to the 84 hospital admissions during the pre-pandemic years, the number of aSAH hospitalizations (*n* = 56) declined during the pandemic without reaching significance. No significant difference in the analyzed patient characteristics including clinical condition at onset, treatment, complications, and outcome, between 56 patients with aSAH admitted during the COVID pandemic and the treated patients in the last 5 years in the pre-COVID period were found. In our multivariable analysis, we detected young age (*p* < 0.05; OR 4.2) and no existence of early hydrocephalus (*p* < 0.05; OR 0.13) as important factors for a favorable outcome (mRS ≤ 0–2) after aSAH during the COVID pandemic. A past COVID-infection was detected in young patients suffering from aSAH (Age <50years, *p* < 0.05; OR 10.5) with an increased rate of cerebral vasospasm after aSAH onset (*p* < 0.05; OR 26). Nevertheless, past COVID-infection did not reach significance as a high-risk factor for unfavorable outcomes.

**Conclusion:**

There was a relative decrease in the number of patients with aSAH during the COVID-19 pandemic. Despite the extremely different conditions of hospitalization, there was no impairing significant effect on the treatment and outcome of admitted patients with aSAH. A past COVID infection seemed to be an irrelevant limiting factor concerning favorable outcomes.

## Introduction

The COVID-19 pandemic caused significant disruption to established care paths, including acute conditions such as aneurysmal subarachnoid hemorrhage (SAH) (aSAH). In March 2020, Germany has declared a state of emergency and implemented restrictions on business, travel, and social life.

In the case of the healthcare system, the main aim was to accommodate the care of critically ill patients with severe acute respiratory syndrome coronavirus 2 (SARS-CoV-2) infection worldwide by rationing of resources (1). Therefore, emergency medical services were changed to conserve resources and mitigate infection risk to patients and their providers (2, 3).

Additionally, cardiac symptoms were reported in some of the most seriously ill COVID-19 patients (4). There was growing recognition that COVID-19 may not be confined to the respiratory system. Further side effects as neuro-invasive features leading to devastating ischemic or hemorrhagic events were related to SARS-CoV-2 infection (5). A raised incidence of strokes in younger patients with COVID-19 has been reported (5).

However, there is a lack of information on the impact of the COVID-19 pandemic on SAH admissions. Early regional or single-center reports from Paris (6) and Toronto (7) suggest a decrease in the number of patients suffering from aSAH, whereas no changes were seen in Berlin (8). Here, we evaluated the impact of COVID-19 on the volumes of aSAH admissions and treatments for patients with ruptured intracranial aneurysms during the peak of the pandemic, defined from March 2020 to February 2021. Here, we aim to quantify the impact of the COVID-19 pandemic on the timing of presentation of all patients with acute aSAH, their treatment management, and progression of the disease.

## Methods

### Clinical Data

We retrospectively analyzed our institutional database of consecutive patients suffering aSAH during the first COVID pandemic year (03/20-02/21), the pre-pandemic year (03/19-02/20), and the last 5 years pre-COVID pandemic (2015-02/2020) as the baseline data. aSAH was defined as a spontaneous non-traumatic hemorrhage into the subarachnoid space with evidence of at least an intracranial aneurysm.

The retrospective clinical study was approved by the local ethics committee of the Goethe University and was performed in accordance with the related guidelines of the regional ethics committee in Frankfurt am Main, Germany. Because of the retrospective design, patient consent form was not needed.

All patients with aSAH, diagnosed by SAH pattern on CT-scan, or confirmed by lumbar puncture, underwent cerebral digital subtraction angiography (DSA) to rule out intracranial vascular bleeding sources. Patients in whom the bleeding source was detected to be an aneurysm were included in this study. Patients with ruptured aneurysms were treated by surgical or endovascular aneurysm occlusion based on an interdisciplinary consensus. Patients not receiving treatment at SAH onset because of advanced brain injury or without clinical follow-up 6 months post-SAH were excluded.

All parameters relevant to this analysis, including patient characteristics such as age, gender, relevant previous diseases in the history, anticoagulation, nicotine abuse, positive family history, active/ past COVID infection, clinical condition at admission using Hunt & Hess classification, bleeding pattern described by Fisher score, treatment procedure “clip vs. coil,” the occurrence of cerebral vasospasm (CVS), delayed cerebral infarction (DCI), delayed ischemic neurological deficit (DIND), early hydrocephalus, shunt implantation, and finally, clinical outcome (modified Rankin Scale: mRS after 6 months; favorable outcome mRS 0–2 vs. unfavorable outcome mRS 3–6) in patients with aSAH, were recorded.

Early hydrocephalus was defined as the external ventricular drain (EVD) placement during the first 24 h after admission because of the neurological decline of patients with aSAH.

The CVS diagnosis in patients was uniformly defined by CT-Angiography as radiological imaging on the basis of arterial narrowing (e.g., >33% mild CVS, 33–66% moderate CVS, >66% severe CVS).

To assess the modified Rankin Scale 6 months after aSAH onset, which is integrated into our standard clinical evaluation forms for aSAH patients, we followed up the course of the disease in all outpatients and still hospitalized patients. In the case of still hospitalized aSAH patients, follow-up data were assessed using unified evaluation forms by rehabilitation clinics and/or nursing homes.

### Statistical Analysis

Data analyses were performed using the computer software package (IBM SPSS, version 22, IBM SPSS Inc.; Armonk, NY, USA). Analyses of categorical variables were done by using Fisher's exact test and GraphPad Prism (8.0, GraphPad Software Inc., USA). Normally distributed variables were expressed as mean values with SD and analyzed using a two-tailed *t*-test. Multivariable analysis was carried out to identify factors for outcome improvement among above-mentioned aspects. A *p* < 0.05 was considered statistically significant.

## Results

### Neuro-Emergency Admission and Characteristics of ASAH Patients Before vs. During COVID-19 Pandemic

**Figure 1:**
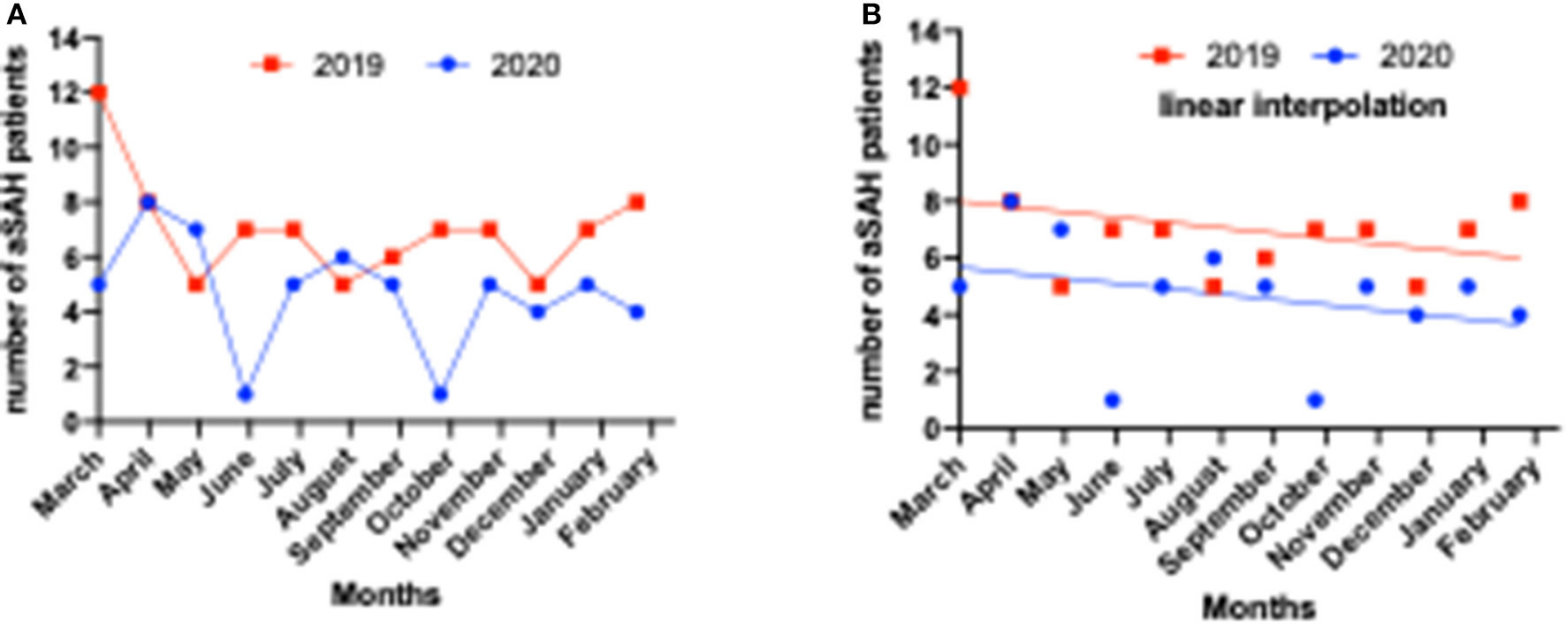
Number of intensive care unit (ICU) – emergency admissions in patients with subarachnoid hemorrhage (SAH) during 1 year of COVID-19 pandemic (2020/2021) vs. the pre-pandemic year (2019/2020) in Frankfurt am Main, Germany. **(A)** Development of the number of patients presenting with aneurysmal subarachnoid hemorrhage per calendar month during COVID-19 pandemic (2020/2021) vs. the pre-pandemic year (2019/2020). **(B)** Incidence of patients admitted because of aneurysmal SAH (aSAH) during 1 year of COVID-19 pandemic (2020/2021) vs. the pre-pandemic year (2019/2020) without reaching a significant difference.

From March 2019 to February 2020, designated the pre-COVID period, 84 patients presented with a final diagnosis of aSAH. From March 2020 to February 2021, defined as the COVID period in our analysis, 56 patients with aSAH were available. The number of cases recorded by every month of the year showed a general decreasing trend in 2020. In this first year of the COVID period, we observed a drop in the absolute number of cases per calendar month without resulting in a statistical significance (Figures 1A,B, Table 1).

**Table 1:** Characteristics of patients with aneurysmal subarachnoid hemorrhage (SAH) (aSAH) during 1 year of COVID-19 pandemic (03/20-02/21) vs. the pre-pandemic year (3/19-02/20) in Frankfurt am Main, Germany.

**Patient characteristics**	**Admission during COVID**	**Admission before COVID**	*p* ^*^	OR^*^
	**pandemic 03/20-02/21**	**pandemic 03/19-02/20**		
Number of patients	56	84	-	-
Place of residence in Frankfurt	7 (13%)	20 (24%)	NS	NS
Distance of residence from Hospital in km ^#^	37 ± 22	41 ± 33	NS	-
Female	36 (64%)	55 (65%)	NS	NS
Age <50 yrs	12 (21%)	19 (23%)	NS	NS
Hypertension	17 (30%)	36 (43%)	NS	NS
Nikotin abuse	22 (39%)	25 (30%)	NS	NS
Anticoagulation	4 (7%)	8 (10%)	NS	NS
Positive family history	2 (4%)	7 (8%)	NS	NS
Delayed hospital admission in days ^#^	0.94 ± 1.45	0.77 ± 1.3	NS	-
Hunt & Hess ≤ III	37 (66%)	55 (65%)	NS	NS
Fisher 3 blood pattern	22 (39%)	47 (56%)	NS	NS
Clipping	20 (36%)	33 (39%)	NS	NS
Coiling	36 (64%)	51 (61%)	NS	NS
Cerebral vasospasm	33 (59%)	54 (64%)	NS	NS
At admission	8 (24%)	11 (20%)	NS	NS
Early CVS onset (d 1-4 postSAH)	11 (33%)	13 (24%)	NS	NS
CVS onset (d 4-8 postSAH)	6 (18%)	11 (20%)	NS	NS
Late CVS onset (d 9 postSAH)	8 (24%)	19 (35%)	NS	NS
CVS duration in days ^#^	8,8 ± 7,7	8,7 ± 8	NS	-
DCI	37 (66%)	49 (58%)	NS	NS
DIND	25 (45%)	45 (54%)	NS	NS
Early hydrocephalus	30 (31%)	55 (65%)	NS	NS
Shunt-implantation	11 (20%)	15 (18%)	NS	NS
Favorable outcome (mRS ≤ 0-2)	18 (32%)	31 (37%)	NS	NS

* *Favorable outcome 6 months after SAH: mRS ≤ 2 points; Unfavorable outcome: mRS >2 points. Data are shown in n (%); Fisher exact test;*

# unpaired t-test; ^*^p < 0,05 is significant. Odd ratio (OR) data with 95% confidence interval.

In spite of the decrease in the number of admitted aSAH patients during the first year of COVID pandemic, there is still a parallel distribution as demonstrated in Figures 1 A,B.

Comparing patient characteristics of admitted patients in the first year of the pandemic with the pre-pandemic year, there is no significant difference according to aSAH risk factors such as age, female gender, hypertension, nicotine abuse, positive family history, and anticoagulation, respectively. There was additionally no significant difference between the severity of presentation, described as Hunt & Hess classification and Fisher blood pattern score, and/or treatment options. Furthermore, a significantly higher risk for aSAH followed complications, such as CVS, DCI, DIND, early hydrocephalus, and related shunt implantation, could not be detected. However, there was no sign of a significantly higher risk for unfavorable outcomes in the case of patients with aSAH admitted during the COVID pandemic (Table 1).

As the neurosurgical Department of University Hospital Frankfurt offering intensive care unit (ICU)-emergency capacity for the Frankfurt Rhein-Main area (ca. 147755 km^2^) (9) the mean time interval between aSAH onset and presentation to our vascular center was 0.77 ± 1.3 days for the pre-COVID, and 0.94 ± 1.45 days for the COVID period, without reaching a statistical significance. Furthermore, the distance of residence of the admitted patients from our center in km showed no significant change (Table 1).

To underline the above-mentioned results, we analyzed patient characteristics of included 56 patients during the COVID pandemic in comparison with admitted aSAH patients to our department during the last 5 pre-COVID years (03/2015-02/2020).

In consistency with the previous results, we obtained no significant difference in the added analyzed factors as patient characteristics including clinical condition at onset, treatment, complications, and outcome, between patients with aSAH admitted during the COVID pandemic and treated patients with aSAH in the last 5 years at our department as pre-COVID period.

Comparing a bigger patient cohort as the pre-pandemic group, there was a single significant higher rate of nicotine abuse in patients suffering aSAH during the pandemic year (Table 2).

**Table 2:** Characteristics of patients with aSAH during 1 year of COVID-19 pandemic (03/20-02/21) vs. the 5 pre-pandemic years (03/15-02/20) in Frankfurt am Main, Germany.

**Patient characteristics**	**Admission during COVID**	**Admission before COVID**	*p* ^*^	OR^*^
	**pandemic 03/20-02/21**	**pandemic 03/15-02/20**		
Number of patients	56	189	-	-
Place of residence in Frankfurt	7 (13%)	47 (25%)	NS	NS
Distance of residence from hospital in km ^#^	37 ± 22	47 ± 29	NS	-
Female	36 (64%)	132 (70%)	NS	NS
Age <50 yrs	12 (21%)	55 (29%)	NS	NS
Hypertension	17 (30%)	74 (39%)	NS	NS
**Nikotin abuse**	**22 (39%)**	**43 (23%)**	**0.02**	**2.2 (1.1–4.0)**
Anticoagulation	4 (7%)	23 (12%)	NS	NS
Positive family history	2 (4%)	10 (5%)	NS	NS
Delayed hospital admission in days ^#^	0.94 ± 1.45	0.70 ± 1.53	NS	-
Hunt & Hess ≤ III	37 (66%)	112 (59%)	NS	NS
Fisher 3 blood pattern	22 (39%)	98 (52%)	NS	NS
Clipping	20 (36%)	82 (43%)	NS	NS
Coiling	36 (64%)	107 (57%)	NS	NS
Cerebral vasospasm	33 (59%)	121 (64%)	NS	NS
At admission	8 (24%)	24 (20%)	NS	NS
Early CVS (d 1-4)	11 (33%)	36 (30%)	NS	NS
CVS (d 4-8)	6 (18%)	30 (24%)	NS	NS
Late CVS (since d 9)	8 (24%)	31 (26%)	NS	NS
CVS duration in days ^#^	8,8 ± 7,7	10,2 ± 8,2	NS	-
DCI	37 (66%)	113 (60%)	NS	NS
DIND	25 (45%)	106 (56%)	NS	NS
Early hydrocephalus	30 (31%)	112 (59%)	NS	NS
Shunt-implantation	11 (20%)	55 (29%)	NS	NS
Favorable outcome (mRS ≤ 0–2)	18 (32%)	89 (47%)	NS	NS

* *Favorable outcome 6 months after SAH: mRS ≤ 2 points; Unfavorable outcome: mRS >2 points. Data are shown in n (%); Fisher exact test;*

# unpaired t-test; ^*^p < 0,05 is significant. Odd ratio (OR) data with 95% confidence interval.

A total of 8 patients (pre-COVID-pandemic cohort: *n* = 7; COVID-pandemic cohort: *n* = 1) could not be included in this study because of hypoxic brain damage diagnosed on the first CT scan at admission as a result of a high volume of SAH and/or followed out of hospital resuscitation. In addition, we excluded 12 patients (pre-COVID-pandemic cohort: *n* = 10; COVID-pandemic cohort: *n* = 2) because of missing follow-up data.

### Characteristics of ASAH Patients With Previous COVID-19 Infection

Among the 56 treated patients with aSAH admitted during the COVID-pandemic to our department, we detected a total of 6 patients (11%) with a past COVID infection in their history. Active infection was not proven in these patients using the SARS-CoV-2-Gen PCR test at admission. None of these patients was vaccinated at the time of hospital admission.

Comparing the patients with past COVID-infection with the non-COVID-infection group suffering from aSAH, we established a significantly higher rate of young patients with post-COVID-infection (Age <50 years, *p* < 0.05; OR 10.5). Additionally, patients with aSAH with past COVID-infection had an increased rate of CVS during days 4–8 after aSAH onset (*p* < 0.05; OR 26). There was no significant delay in hospital admission in days in the case of these patients. Both patient groups had the same chance for favorable outcomes (Table 3). None of the patients with past COVID-infection was impaired by respiratory dysfunction.

**Table 3:** Characteristics of patients with aSAH during 1 year of COVID-19 pandemic (03/20-02/21) w/o previous COVID Infection.

**Patient characteristics**	**COVID infection**	**Non-COVID infection**	*p* ^*^	OR^*^
Number of patients	6	50	-	-
Female	4 (67%)	32 (64%)	NS	NS
**Age** **<** **50 yrs**	**4 (67%)**	**8 (16%)**	**0.02**	**10.5(2.0–58.6**)
Hypertension	2 (33%)	15 (30%)	NS	NS
Nikotin abuse	3 (50%)	19 (38%)	NS	NS
Anticoagulation	2 (33%)	2 (4%)	NS	NS
Positive family history	0 (0%)	2 (4%)	NS	NS
Delayed hospital admission in days ^#^	1 ± 1,33	0,88 ± 1,42	NS	-
Hunt & Hess ≤ III	3 (50%)	34 (68%)	NS	NS
Fisher 3 blood pattern	4 (67%)	18 (36%)	NS	NS
Clipping	3 (50%)	17 (34%)	NS	NS
Coiling	3 (50%)	33 (66%)	NS	NS
Cerebral vasospasm	4 (67%)	29 (58%)	NS	NS
At admission	0 (0%)	8 (16%)	NS	NS
Early CVS (d 1-4)	1 (17%)	10 (20%)	NS	NS
**CVS (d 4-8)**	**3 (50%)**	**3 (6%)**	**0,02**	**26(2.6–343)**
Late CVS (since d 9)	0 (0%)	8 (16%)	NS	NS
CVS duration in days	9.5 ± 7.1	8.8 ± 7.7	NS	-
DCI	3 (50%)	34 (68%)	NS	NS
DIND	4 (67%)	21 (42%)	NS	NS
Early hydrocephalus	3 (50%)	27 (54%)	NS	NS
Shunt-implantation	2 (33%)	9 (18%)	NS	NS
Favorable outcome (mRS ≤ 0–2)	2 (33%)	16 (32%)	NS	NS

* *Favorable outcome 6 months after SAH: mRS ≤ 2 points; Unfavorable outcome: mRS >2 points. Data are shown in n (%); Fisher exact test;*

# unpaired t-test; ^*^p < 0.05 is significant. Odd ratio (OR) data with 95% confidence interval.

Using a multivariable analysis to verify significant factors for a favorable outcome (mRS ≤ 0–2) after aSAH during the COVID pandemic, we detected young age (*p* < 0.05; OR 4.2) and no existence of early hydrocephalus at the initial aSAH onset (*p* < 0.05; OR 0.13) as important factors. A past COVID-infection, which increased the rate of CVS post aSAH in Table 3, did not reach significance as a high-risk factor (Table 4).

**Table 4:** Clinical outcome in association with patient characteristics in patients with aSAH during 1 year of COVID-19 pandemic (03/20-02/21) w/o previous COVID Infection.

**Patient characteristics**	Favorable outcome (mRS ≤0–2)^*^	**Unfavorable outcome (mRS > 2)**	**Multivariate analysis *p* value; OR (95% CI)**
Number of patients	18	38	-
Female	10 (56%)	26 (68%)	NS
**Age** **<** **50 yrs**	**7 (39%)**	**5 (13%)**	**0.04 (OR 4.2; 1.1–15.4)**
Hypertension	6 (33%)	11 (29%)	NS
Nikotin abuse	7 (39%)	15 (39%)	NS
Anticoagulation	3 (17%)	1 (3%)	NS
Positive family history	0 (0%)	2 (5%)	NS
Hunt & Hess ≤ III	15 (83%)	22 (58%)	NS
Fisher 3 blood pattern	4 (22%)	18 (47%)	NS
Clipping	5 (28%)	15 (39%)	NS
Coiling	13 (72%)	23 (61%)	NS
Cerebral vasospasm	11 (61%)	22 (58%)	NS
DCI	10 (56%)	27 (71%)	NS
**Early hydrocephalus**	**4 (22%)**	**26 (68%)**	**0.002 (OR 0.13; 0.04–0.5)**
COVID infection	2 (11%)	4 (11%)	NS

* *Favorable outcome 6 months after SAH: mRS ≤ 2 points; Unfavorable outcome: mRS >2 points. Data are shown in n (%); Fisher exact test; p <0.05 is significant. Odd ratio (OR) data with 95% confidence interval*.

## Discussion

### Impact of COVID-19 Pandemic on Treatment Management of ASAH Patients

Our neurosurgical department offers emergency neurosurgical coverage in Frankfurt and suburbs for a population of approximately 5.816,186 inhabitants (9).

The German Government introduced mitigation measurements in March 2020 to drastically limit social interactions and consequently virus diffusion. As noted in the result section, we described a decrease of acute aneurysmal SAH as a leading vascular center in the region.

Concerning the decreased number of admitted patients with aSAH, a robust association has been found between psychological stress and aneurysm rupture risk (10). The potential mechanisms behind the association between perceived stress and an increased risk of aSAH are complex and yet not fully understood. The overstimulation of the hypothalamus-pituitary-adrenal axis and increased release of cortisol are related as possible mechanisms. In addition, acute as well as chronic psychosocial stress is also associated with endothelial dysfunction (11). Several risk factors for rupture of intracranial aneurysms were defined, including a sudden increase in blood pressure, which may be stress-induced (12). The precited factors should probably explain an increase and not a decrease of SAH as we observed during clinical practice. The possible explanations to such epidemiological situation defined by a Parisian group (6) are decrease of people seeking medical help fearing to get infected, high load on healthcare system resulting in misdiagnosis, especially for headache as a common symptom of COVID infection (13), and some still unknown deaths of quarantined people.

Bernat et al. (6) also described patients that experienced aneurysm rupture at risk of rebleeding as a fragile population of patients, who are part of the “collateral damages” of the COVID pandemic.

Experiencing a model of a central COVID ICU at the University Hospital of Frankfurt to treat all COVID infected patients and further discipline-specific non-COVID ICUs to take care of non-COVID emergencies, approximately all hospital transfer requests of patients with aSAH from external regional hospitals without neurosurgical department or Neuro-ICU capacity could be realized during the COVID period.

We also detected a decrease in the number of presented patients with aSAH in our department compared to the pre-pandemic year, which could be the result of the already above-mentioned criteria: not seeking medical help, being afraid of infection, misdiagnosis, and the unknown number of deaths caused by undetected aSAH.

Furthermore, considering the number of admitted patients with aSAH during the last 5 pre-pandemic years, we noticed a variation in the number of patients suffering from aSAH during the years. Therefore, the small difference in the number of admitted patients during the pandemic vs. pre-pandemic year should not be the focus of our evaluation.

Nevertheless, all patients diagnosed with aSAH could be treated after transfer to our ICU being under standard therapy conditions. All these patients had no proof of active COVID and were not infectious. As already demonstrated, there was no significant difference in time-space between clinical onset and admission at our center, which is an important preventive factor in case of aneurysm rebleeding associated with poorer outcomes. In contrast, an association between the outbreak of the COVID-pandemic in the US with a delay in presentation of patients was reported repeatedly as a therapeutic limitation in case of patients with acute ischemic stroke, which had a strong negative effect on treatment management and neurological outcomes (14–16).

This was already reported in a big international cohort in case of subarachnoid hemorrhage describing the decrease in SAH volumes, including the embolization of ruptured aneurysms, similar to reports of decreases in stroke admissions, intravenous thrombolysis, MT, and acute ST-elevation myocardial infarction (STEMI) activations during the COVID-19 pandemic (14, 17, 18).

Fortunately, there was no proof of significant delay in hospital admission in our department, providing patients from the same residence area as pre-COVID pandemic time. Furthermore, we could not detect any significant differences in clinical complications and outcomes after SAH comparing the pandemic SAH patient group with pre-pandemic treated ones. This aspect is the proof for the continuation of evidence-based treatment management during COVID-pandemic including immediate external ventricular drainage to decrease the high intracerebral pressure, DSA during the first 24 h after SAH onset, and treatment by clipping or coiling in interdisciplinary dialogue.

However, to care for the massive numbers of patients with COVID, many hospital systems and surgeons as our department were focused on conserving resources by limiting elective surgical procedures. During the peak of the pandemic in Western Europe, COVID-19 disrupted the practice of neurosurgeons and affected the decision-making in triaging neurosurgical cases. A majority of surgeons reported that all elective cases and clinics were re-scheduled (2, 8, 19).

This aspect encouraged the Neuro-ICU capacity to treat neurological emergencies such as subarachnoid hemorrhage.

Interestingly, there was a single significant higher rate of nicotine abuse in patients suffering aSAH during the pandemic year compared to the data from the last pre-pandemic years. Interpreting smoking as a misconduct could be related to the psychological stress caused by being quarantined as a result of the pandemic (20).

A total of 8 patients (pre-COVID-pandemic cohort: *n* = 7; COVID-pandemic cohort: *n* = 1) could not be included in this study because of hypoxic brain damage diagnosed on the first CT scan at admission as a result of a high volume of SAH and/or followed out of hospital resuscitation. In addition, we excluded 12 patients (pre-COVID-pandemic cohort: *n* = 10; COVID-pandemic cohort: *n* = 2) because of missing follow-up data. The aim of this analysis is to evaluate and compare the medical care and treatment of patients with aSAH before and during the COVID pandemic and the course of the disease, as well as the clinical outcome of these patients, including a small number of patients with incomplete target database, was not conducive to reaching the goal of this study.

### Effect of Past COVID Infection on the Progression of ASAH

Detecting six aSAH patients who had a past mild COVID infection in the history without respiratory dysfunction, we compared the progression of the disease in these patients with the non-COVID SAH group treated during the same time-space. This study is not designed to establish causality between COVD-19 and cerebral aneurysm rupture; instead, we tried to describe and analyze the patient characteristics in patients with past COVID infections. Because of the high incidence of aneurysm ruptures in young patients in this series, the inflammatory response accompanying COVID-19 should be under consideration as a cause of premature rupture of preexisting cerebral aneurysms. Based on recent data, any relationship between SARS-CoV-2 infection and cerebral aneurysm rupture could possibly involve macrophage-mediated production of interleukin-1β, interleukin-6, and tumor necrosis factor-α. Macrophages are highly implicated in both, aneurysmal rupture and COVID-19–related inflammations (21–24).

The point that these patients had a significantly higher risk for CVS day 4–8 after SAH underlined the fact that macrophage-mediated vasospasm could be encouraged by COVID-related inflammation (25). This remains, however, purely speculative and warrants additional investigation. A larger sample of patients with aSAH and measurement of inflammatory mediators would be especially valuable in defining the association between SARS-CoV-2 infection and aSAH.

On the other hand, the increased rate of CVS during day 4–8 after aSAH onset in patients with past COVID-infection (*p* < 0.05; OR 26) could be reasonable due to the higher number of patients with worse admission status and more often high Fisher grade.

Nevertheless, talking with the young patients with aSAH with post-COVID infection in this group, one should consider the higher risk of vasospasm in young patients after aSAH, which has already been described in previous datasets (26, 27). Therefore, a premature evaluation of these sparse data sources should not mislead further clinical trials on this field.

However, using multivariate analysis to detect significant factors to reach a favorable outcome in our COVID pandemic group, past COVID infection was not proved as a limitation factor, but higher age > 50 years and early hydrocephalus. This also should be studied in further studies including a higher number of patients to obtain a relevant response to this question.

### Study Limitations

The rapidly changing landscape of the COVID-19 pandemic, including the effects of social distancing, may not yet be captured in this dataset. We do not analyze the difference in referral patterns and patient flows between different centers in this study. We assume the efficiency of the stroke referral system of the center and stable conditions between the baseline and the COVID period.

Pointing to the limitations of the study, the retrospective design and the small number of detected patients with post-COVID SAH should be mentioned. In addition, detecting six patients suffering from aSAH after a COVID infection could not be sufficiently powered by statistical analysis to prove the clinical impact on these patients with aSAH. Nevertheless, a separated multivariable analysis of the small patient group with past COVID infection detected a tenuous significantly higher risk for CVS as a limiting factor during the progression of the disease, surprisingly without affecting the chance of favorable outcome (Tables 3, 4)

However, further prospective clinical trials should be spent on the epidemiological aspect and clinical impairment of COVID-pandemic in young patients affected by aneurysmal subarachnoid hemorrhage. The next steps should be designed as multicenter clinical trials.

## Conclusion

These data demonstrate decreasing hospital admissions due to aneurysmal subarachnoid hemorrhage despite unlimited hospital resources for acute stroke care. We suggest that this may be a consequence of social distancing measures. Hence, raising public awareness is necessary to avoid serious healthcare and economic consequences of undiagnosed and untreated diseases. Nevertheless, this current work shows a successful model of health care management in the case of SAH as an acute neurological emergency without any disadvantage for the patients.

Over 1 year into the COVID-19 pandemic, the global community is constantly discovering sequelae of SARS-CoV-2 infection. In this cohort, we report six aSAH cases with past COVID-infection. These cases are one of the first reported patients of this specific phenomenon and they raise questions on a possible interaction between COVID infection and cerebral aneurysm rupture and clinical outcome after aSAH. The young age of detected patients and the high risk for CVS, deserve further basic research.

## Data Availability Statement

The original contributions presented in the study are included in the article/supplementary material, further inquiries can be directed to the corresponding author.

## Ethics Statement

The studies involving human participants were reviewed and approved by Ethic Committee, Medical Department, University Hospital Frankfurt, Goethe-University, Theodor-Stern-Kai 7., 60590 Frankfurt am Main, Germany. Written informed consent for participation was not required for this study in accordance with the national legislation and the institutional requirements.

## Author Contributions

SK, LQ, NB, and FK contributed the data. SK wrote the main manuscript text and prepared Figure 1 and Tables 1–4. All authors reviewed the manuscript. All authors contributed to the article and approved the submitted version.

## Conflict of Interest

The authors declare that the research was conducted in the absence of any commercial or financial relationships that could be construed as a potential conflict of interest.

## Publisher's Note

All claims expressed in this article are solely those of the authors and do not necessarily represent those of their affiliated organizations, or those of the publisher, the editors and the reviewers. Any product that may be evaluated in this article, or claim that may be made by its manufacturer, is not guaranteed or endorsed by the publisher.
